# Role of the indoleamine-2,3-dioxygenase/kynurenine pathway of tryptophan metabolism in behavioral alterations in a hepatic encephalopathy rat model

**DOI:** 10.1186/s12974-017-1037-9

**Published:** 2018-01-04

**Authors:** Xi Jiang, Lexing Xu, Lin Tang, Fuhe Liu, Ziwei Chen, Jiajia Zhang, Lei Chen, Cong Pang, Xuefeng Yu

**Affiliations:** 1 Department of Pharmacy, Zhejiang Pharmaceutical College, Ningbo, Zhejiang Province 315000 China; 2 Department of Neurosurgery, Huai’an First People’s Hospital, Nanjing Medical University, Nanjing, Jiangsu Province 223001 China; 3 Mingzhou Hospital, Zhejiang University, Hangzhou, Zhejiang Province 315000 China

**Keywords:** Hepatic encephalopathy (HE), Inflammation, Indoleamine-2,3-dioxygenase (IDO), Serotonin (5-HT), Depression, Anxiety

## Abstract

**Background:**

This study aims to explore the role of indoleamine-2,3-dioxygenase (IDO)/kynurenine (KYN) pathway of tryptophan (TRY) metabolism in behavioral alterations observed in hepatic encephalopathy (HE) rats.

**Methods:**

Expression levels of proinflammatory cytokines were tested by QT-PCR and ELISA, levels of IDOs were tested by QT-PCR and Western blot, and levels of 5-hydroxytryptamine (5-HT), KYN, TRY, 3-hydroxykynurenine (3-HK), and kynurenic acid (KA) in different brain regions were estimated using HPLC. Effects of the IDO direct inhibitor 1-methyl-l-tryptophan (1-MT) on cognitive, anxiety, and depressive-like behavior were evaluated in bile duct ligation (BDL) rats.

**Results:**

Increased serum TNF-α, IL-1β, and IL-6 levels were shown in rats 7 days after BDL, and these increases were observed earlier than those in the brain, indicating peripheral immune activation may result in central upregulation of proinflammatory cytokines. Moreover, BDL rats showed a progressive decline in memory formation, as well as anxiety and depressive-like behavior. Further study revealed that IDO expression increased after BDL, accompanied by a decrease of 5-HT and an increase of KYN, as well as abnormal expression of 3-HK and KA. The above results affected by BDL surgery were reversed by IDO inhibitor 1-MT treatment.

**Conclusion:**

Taken together, these findings indicate that (1) behavioral impairment in BDL rats is correlated with proinflammatory cytokines; (2) TRY pathway of KYN metabolism, activated by inflammation, may play an important role in HE development; and (3) 1-MT may serve as a therapeutic agent for HE.

**Electronic supplementary material:**

The online version of this article (10.1186/s12974-017-1037-9) contains supplementary material, which is available to authorized users.

## Background

Hepatic encephalopathy (HE) is a neuropsychiatric abnormality in patients with acute or chronic liver disease. It is characterized by personality mood change, cognitive impairment, decreased consciousness, and coma [1, 2]. The development of HE negatively impacts patient survival, and the survival reports of patients with HE showed a survival rate of 42% after 1 year and 23% after 3 years [3, 4]. Such high mortality in HE patients is due to the lack of effective treatment method in clinic. Therefore, it is urgent to identify potential therapeutic target for HE.

Neurological alteration in HE was affected by both hyperammonemia and inflammation [5, 6]. The serum and brain levels of proinflammatory cytokines, such as IL-1β, IL-6, and TNF-α, are higher in HE patients than those in patients only suffer from liver disease [6, 7]. Hyperammonemia and inflammation act synergistically to induce depression, anxiety, and cognitive impairment, as shown in the research analyzing neurological impairment in HE patients with different degrees of inflammation and hyperammonemia [8]. Furthermore, it was found that the inflammation induced by HE is most obvious in the hippocampus and cerebral cortex, two important brain regions regulating executive functioning, working memory, and motor planning [9–11].

Recently, kynurenine (KYN) pathway, which regulates tryptophan (TRY) metabolism and the serotonergic system, was hypothesized to be important in mediating the effects of proinflammatory cytokines on the brain [12–14]. Normally, TRY is metabolized to KYN by indoleamine-2,3-dioxygenase (IDO)1, IDO-like enzyme, IDO2, and tryptophan-2,3-dioxygenase (TDO), then KYN is metabolized to kynurenic acid (KA, Fig. 11), an *N*-methyl-d-aspartate receptor (NMDAR) antagonist [15, 16]. TRY also can be metabolized to serotonin or 5-hydroxytryptamine (5-HT), which is further broken down by monoamine oxidase (MAO) to 5-hydroxyindoleacetic acid (5-HIAA). However, proinflammatory cytokines upregulate the expression of IDO with inflammation status, resulting in the activation of another metabolic pathway of KYN. In this circumstance, KYN is more likely to be metabolized to quinolinic acid (QA), a neurotoxic metabolite [17, 18]. Moreover, activation of IDO shifts TRY metabolism from serotonin synthesis to KYN formation, inducing an imbalance of the serotonergic system [19]. This disorder of KYN pathway is hypothesized to underlie inflammation-associated depression and anxiety behaviors [20]. Previous studies have proved that activation of IDO and high QA expression were found in an animal model of depression, and treatment with an IDO inhibitor prevented the development of inflammation-induced depressive-like behaviors in rodents [21–23]. High levels of QA were also observed in the brain and serum of HE animal model by Basile et al. [24].

Although inflammation-induced changes in KYN metabolism have been largely studied in depression and anxiety, pathogenesis of HE regarding the KYN pathway of TRY metabolism was never investigated. Therefore, the present study aims to assess whether inflammation-induced changes of KYN pathway contribute to depressive-like symptoms, anxiety-like symptoms, and cognitive impairment in rats with HE to seek possible therapeutic target for HE.

## Methods

### Animals

Male Wistar rats (220–240 g) were obtained from the Animal Center of Shanghai Branch, Chinese Academy of Sciences. Upon arrival, the rats were housed six per cage (54 × 39.5 × 20 cm) and acclimatized to a colony room with controlled ambient temperature (22 ± 1 °C), humidity (50 ± 10%), and a 12-h natural light/dark cycle. They were acclimatized to the local vivarium for a week prior to experiment. All the procedures were approved by the Wenzhou Medical University Committee on Animal Care and Use and were conducted in accordance with the guidelines for humane use and care of laboratory animals. The experiments were designed for a duration of 4 weeks, and rats were randomly segregated into five groups (0, 7, 14, 21, and 28 days). Each group contains five subgroups (*n* = 16 in each subgroup): sham (rats received identical laparotomy without bile duct ligation (BDL), used as control), BDL (rats underwent BDL surgery, model set), and BDL + 1-methyl-l-tryptophan (1-MT) (dose in 1, 3, and 9 mg/kg) groups. In this study, some rats died during the surgical process; a total of 450 rats were used to ensure that each subgroup contains at least 16 rats in data analysis.

### Treatment schedule

**Fig. 1:**
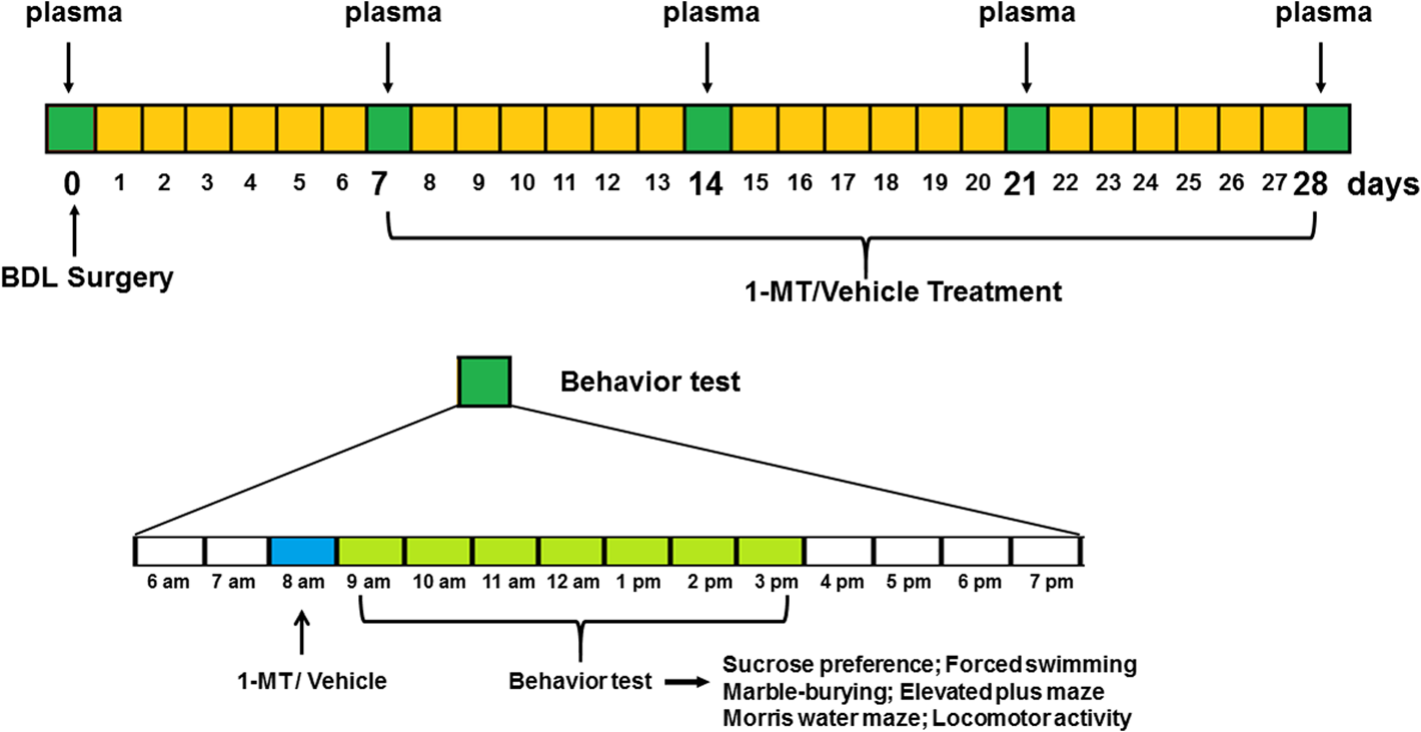
Scheme showing the experimental design. Blood was taken 0, 7, 14, 21, and 28 days after BDL surgery. Rats received the first administration of 1-MT (1, 3, and 9 mg/kg, p.o.) or vehicle at the 7th day after BDL surgery, and behavior tests were performed at 0, 7, 14, 21, and 28 days after BDL surgery. Animals were sacrificed immediately after behavior tests for neurochemical analysis

All the rats received BDL surgery except the sham group. After they recovered for 7 days, the first administration of 1-MT (Sigma-Aldrich) was conducted. Before administration, 1-MT powder was firstly dissolved in alkaline water (vehicle) (pH 11.0) to prepare 1, 3, and 9 mg/ml 1-MT solutions. One millilitre per kilogram (body weight) of 1-MT solution was intraperitoneally injected into each rat. BDL + 1-MT rats received administration of 1-MT (dose in 1, 3, and 9 mg/kg) from the 7th day to the 28th day (each day at 8 a.m.). Sham and BDL rats were injected with alkaline water instead. Each animal received behavioral test 0, 7, 14, 21, and 28 days after BDL surgery. The experimental design is summarized in Fig. 1. For performing multiple tests on the same rat may cause inaccurate results, each subgroup was divided into two sets (*n* = 8 per set). Animals in one set were used for sucrose preference test, forced swimming test, marble-burying test, and elevated plus maze test to study anxiety and depressive-like behavior. Animals in another set were used for Morris water maze assay. All the rats received locomotor activity test. After behavioral tests, rats were sacrificed 0 day (before BDL surgery) and 7, 14, 21, and 28 days after surgery. The hippocampus, cerebral cortex, hypothalamus, and striatum were rapidly dissected out and stored at − 80 °C for homogenization. Brain tissues were used in detecting proinflammatory cytokines, IDO expression, and TRY metabolism. When performing the biochemical measurements, animal tissues in one subgroup were also divided into two sets (*n* = 8 per set), which was just the division in the behavior test. Animal brains in one set were used for QT-PCR and Western blot, and animal brains in another set were used for ELISA and HPLC. As for each set, brain tissues in each rat was homogenized and divided into two equal ones, which were used in different biological experiments. Blood was also collected for separation of plasma, which was then stored at − 80 °C. Serum samples were used in liver function test and proinflammatory cytokine determination.

### BDL surgery

Animals were anesthetized by intraperitoneal (i.p.) injection of mixed solution of ketamine hydrochloride (50 mg/kg) plus xylazine (5 mg/kg). Ketamine hydrochloride and xylazine powder were firstly dissolved in alkaline water (vehicle) (pH 11.0) to prepare 2.5 and 1.25 mg/ml solutions, respectively. Proper volume of each solution was extracted according to the exact body weight of each rat and then mixed with another solution before injection. The surgical procedure was performed aseptically according to the study reported by Kountouras (1984). [25] The sham operation consisted of laparotomy and bile duct identification and manipulation without ligation. For rats in the BDL group, the main bile duct was first ligated using two ligatures approximately 0.5 cm apart and then transected at the midpoint between the two ligatures. Immediately after the operation, each animal was placed alone in a cage for 4 h to avoid wound dehiscence and then moved to its original cage. Operative mortality was less than 5%.

### Sucrose preference test

The sucrose preference test (SPT) was carried out as previously described [26]. Before the test, rats were exposed to both test solution (1% sucrose) and tap water for a period of 24 h. During the test, sucrose preference was evaluated for 1 h by utilizing two bottles of 1% sucrose and tap water. The sucrose preference was calculated as the ratio of consumed sucrose solution to the consumed total amount of liquid.

### Forced swimming test

The forced swimming test (FST) has been widely used to identify depressive-like behavior in animals [27, 28]. Briefly, rats firstly underwent a swimming stress session for 15 min (pre-test) in a glass cylinder (height 40 cm; diameter 18 cm; containing 23 cm of water at 24 ± 1 °C). Twenty-four hours later, the rats were placed in the cylinder again for 5 min (test session). The duration that rats remained immobile during a 5-min observation period was recorded. A rat was considered to be immobile when it ceased struggling and remained floating motionless in the water, or made only small movements necessarily to keep its head above the water.

### Marble-burying test

The marble-burying test was carried out as previously described [29]. In brief, each rat was placed individually in a polypropylene cage (40 × 24 × 20 cm) containing nine clean glass marbles (diameter 2.3 cm), which were evenly spaced on sawdust of 5 cm deep. Ten minutes later, rats were removed, and the number of marbles at least one half buried in the sawdust was recorded.

### Elevated plus maze test

The elevated plus maze test was carried out as previously described [30]. In brief, the rat was placed on the apparatus of an elevated platform, which consists of two open arms, two closed arms, and a central platform. At the beginning of the test, the rat was located on the central platform facing open arm. The number of entries and the total time spent in the closed and open arms, respectively, were quantified during a 10-min period using video tracking software (JZZ98; Institute of Materia Medica, Chinese Academy of Medical Sciences, China).

### Morris water maze test

The Morris water maze test was carried out as previously described [31]. The apparatus contains a circular plastic pool (diameter 140 cm; high 60 cm) located in a well-illuminated room with external cues visible from the inside of the pool, which was filled with opaque water (21 ± 1 °C). A hidden circular platform was submerged 2 cm under the water in one of four quadrants.

The acquisition trials (training to escape to the hidden platform) were carried out for six blocks consisting of three trials separated by 20-min inter-block intervals during which the platform remained in the same location relative to the distal cues in the room. On each trial, the rats were placed in the water at different start locations (E, S, W, and N), which were equally spaced from each other and offset from the goal location by 45°. One hour following the sixth block, the hidden platform was removed and the rats were scored during a 60-s probe trial for latency to reach, and crossings over, the previous platform location (memory recall). Another probe trial was run 24 h after training to assess consolidation and retrieval of memory. The escape latency and the number of crossing target quadrant were recorded 0, 7, 14, 21, and 28 days after BDL surgery using video tracking software (JZZ98; Institute of Materia Medica, Chinese Academy of Medical Sciences, China).

### Locomotor activity

The locomotor activity was monitored using an actophotometer [32]. The rat was placed in a square chamber (40 × 40 × 40 cm) which is connected to photoelectric cells with light beams passing through the chamber for 10 min. During this period, the number of light beam breaks was recorded.

### Liver function test

Blood samples were taken from the orbital sinus of animals in each group (7, 14, 21, and 28 days) under light ether anesthesia. Blood was collected and kept for 1 h at room temperature for clotting. Serum was separated by centrifuging the blood sample at 3000 rpm for 20 min. The biochemical parameters, serum alanine aminotransferase (ALT), aspartate aminotransferase (AST), alkaline phosphatase (ALP), ammonia, bilirubin, and total protein content in the serum of each rat, were evaluated using enzyme-linked immunosorbent assay according to the kit protocols (Nanjing Jiancheng Bioengineering Institute, Nanjing, China).

### HPLC

Brain levels of KYN and TRP were determined as previously described [14]. Hippocampus and cerebral cortex tissues of each animal were weighed respectively, and then each tissue sample was mechanically homogenized in 0.1 N HClO_4_ + 25 μM ascorbate (50 μL lysate was added to every 10 mg tissue) using an ultrasonic tissue disruptor. Supernatants were extracted and loaded into a Costar Spin-X centrifuge tube filter (0.22 μM) and centrifuged at 12,000 rpm for 5 min. KYN and TRP were determined by HPLC. Mobile phase (pH = 4.6) consists of 75 mM NaH_2_PO_4_, 25 μM EDTA (disodium salt), and 100 μL/L triethylamine diluted in acetonitrile/water (6:94 *v*/*v*) solution.

The contents of 5-HT, 5-HIAA, 3-hydroxykynurenine (3-HK), QA, and KA in samples were analyzed by HPLC. Frozen brain tissue samples (cerebral cortex and hippocampus) of each animal were homogenized by ultrasonication in 200 μL of 0.4 M perchloric acid (50 μL lysate was added to every 10 mg tissue) and then centrifuged at 12,000 rpm (4 °C) for 20 min. An aliquot of 160 μL supernatant was added to 80 μL solution containing 0.2 M potassium citrate, 0.3 M dipotassium hydrogen phosphate, and 0.2 M EDTA and then centrifuged at 12,000 rpm for 20 min. The mobile phase (pH = 3.0) consists of 75 mM NaH_2_PO_4_, 25 μM EDTA, 0.45 mM octanesulfonic acid, and 100 μL/L triethylamine diluted in acetonitrile/water (6:94 *v*/*v*) solution.

### Quantitative real-time RT-PCR

IL-1β, IL-6, and TNF-α expression levels in the hippocampus, cerebral cortex, hypothalamus, and striatum, and IDO expression in the hippocampus and cerebral cortex of each rat were measured by QT-PCR. Total cellular RNA was isolated using TRIzol reagent (Invitrogen) according to the manufacturer’s protocol, and RNA (1 mg) was reverse transcribed using an MJ Mini™ Gradient Thermal Cycler (Bio-Rad, Hercules CA, USA). RNA concentration was determined at 260 nm using a spectrophotometer (Bio-Rad Labs). The PCR reaction was performed using an iCycler Real-Time PCR machine (Bio-Rad, Hercules CA, USA). Each sample was added with SYBR Green (iQ SYBR Green Supermix reagent, Bio-Rad) at a concentration of 50 nmol/L. The primer sequences were as follows: IL-1β (forward: 5′-TGG ACT TCG CAG CAC AAA ATG-3′; reverse: 5′-GTT CAC TTC ACG CTC TTG GAT-3′), IL-6 (forward: 5′-CCA GAA ACC GCT ATG AAG TTC CT-3′; reverse: 5′-CAC CAG CAT CAG TCC CAA GA-3′), TNF-α (forward: 5′-GCT GGA TCT TCA AAG TCG GGT GTA-3′; reverse: 5′-TGT GAG TCT CAG CAC ACT TCC ATC-3′), IDO (forward: 5′-AGA AGT GGG CTT TGC TCT GC-3′; reverse: 5′-TGG CAA GAC CTT ACG GAC ATC TC-3′), IDO2 (forward: 5′-AAG CTT ATG GAG CCT CAA AGT CAG AGC-3′; reverse: 5′-CTC GAG CTA AGC ACC AGG ACA CAG G-3′), TDO (forward: 5′-TGG GAA CTA GAT TCT GTT CG-3′; reverse: 5′-TCG CTG CTG AAG TAA GAG CT-3′), and β-actin (forward: 5′-TGG AAT CCT GTG GCATCC ATG AAA C-3′; reverse: 5′-AA AAC GCA GCT CAG TAA CAGTCC G-3′). Amplification was performed by initial denaturation at 95 °C for 10 min, followed by 40 cycles at 95 °C for 10 s, 58 °C for 30 s, and 72 °C for 50 s. At the end of the PCR reaction, a melting curve was obtained by holding at 95 °C for 15 s, cooling to 60 °C for 1 min, and then heating slowly at 0.5 °C/s to 95 °C. Melt curve analysis and gel electrophoresis were then conducted to verify the specificity and purity of the PCR products. All the data were normalized to β-actin.

### Western blot analysis

Protein levels of IDO in the hippocampus and cerebral cortex of each rat were measured by Western blot. The tissue samples were firstly weighed and then added with RIPA lysis buffer (Upstate Chemicon, Temecula, CA, USA) (50 μL lysate was added to every 10 mg tissue) and centrifuged at 13,000 rpm for 30 min at 4 °C. Total protein concentrations of the supernatants were assessed using a BCA assay kit (Thermo Scientific, Rockford, IL, USA). Proteins in lysate (40 μg per lane) were resolved using 10% sodium dodecyl sulfate polyacrylamide gel and transferred onto polyvinylidene difluoride membranes. Blots were then incubated in blocking buffer for 2 h at room temperature, washed in Tris-buffered saline with 0.1% Tween 20 (TBST), and incubated with the appropriate primary antibodies over night at 4 °C (anti-IDO, 1:1000, ab106134; anti-TDO2, 1:1000, ab123403; anti-β-actin, 1:1000, ab8227). After washing with TBST, the blots were incubated with the secondary antibodies (1:10,000) for 1 h at room temperature. The detection quantification of specific bands was carried out using a fluorescence scanner (Odyssey Infrared Imaging System, LI-COR Biotechnology, South San Francisco, CA, USA) at 700 and 800 nm wavelengths.

### ELISA

Protein levels of IL-1β, IL-6, and TNF-α in the hippocampus, cerebral cortex, hypothalamus, striatum, and serum were measured by an ELISA kit (R&D Systems China Co., Ltd.). Briefly, serial dilutions of protein standards and samples of each rat were added to ELISA plates, followed by biotinylated anti-IL-1β, IL-6, and TNF-α antibody addition. Then, the prepared solution of avidin, horseradish peroxidase-conjugated complex was added after rinsing with wash buffer. The reaction was stopped by the stopping solution, and absorbance was read at 450 nm [33, 34].

### Statistical analysis

Results were presented as mean ± SEM. All data were statistically analyzed with SPSS software (International Business Machines Corporation, IBM, USA), and carried out by two-way or one-way analysis of variance (ANOVA). For two-way ANOVA, the procedure (sham or BDL surgery) and the treatment (saline vs. 1-MT treatment) were taken as between-group factors. When needed, the time of measurement (0, 7, 14, 21, and 28 days after BDL) was taken as a within-subject factor. The Newman-Keuls test was used for post hoc comparisons. For one-way ANOVA, Dunnett’s test was used for multiple comparisons to determine whether the means differed significantly between two groups. A value of *p* < 0.05 was considered statistically significant.

## Results

### Induction of BDL

Seven days after bile duct ligation, the animals showed signs of cholestasis (jaundice, dark urine, and steatorrhea), which was similar with Eslimi’s finding [35]. ALP, ALT, and AST levels (represent liver function) along with ammonia and bilirubin levels are depicted in Table 1. Activity of ALP, ALT, and AST enzymes was increased by approximately twofold relative to the sham group 7 days after BDL surgery. Moreover, ammonia level in plasma was increased by threefold at the 7th day after BDL. The levels of total, direct (conjugated), and indirect (unconjugated) bilirubin in serum showed approximately fivefold increases in BDL rats.

**Table 1:** Liver function tests in sham and BDL rats

	Sham (0 day)	BDL			
		7 days	14 days	21 days	28 days
ALP (IU/L)	112.4 ± 7.2	217.4 ± 9.1*	274.2 ± 10.2**	289.4 ± 11.2**	327.5 ± 12.8**
ALT (IU/L)	42.5 ± 4.3	79.4 ± 5.2*	91.5 ± 6.4*	97.5 ± 7.4**	110.7 ± 8.7**
AST (IU/L)	120.5 ± 10.1	227.7 ± 11.1*	257.5 ± 12.5*	268.4 ± 11.4*	294.5 ± 15.4**
Ammonia (μmol/L)	21.4 ± 3.2	68.1 ± 5.2*	134.7 ± 8.7**	157.8 ± 9.1**	187.4 ± 10.2***
Total bilirubin (mg/dL)	0.41 ± 0.01	2.5 ± 0.1*	4.6 ± 0.15**	5.2 ± 0.13**	5.9 ± 0.31***
Direct bilirubin (mg/dL)	0.20 ± 0.01	1.4 ± 0.1*	2.1 ± 0.12**	2.4 ± 0.14**	2.7 ± 0.21***
Indirect bilirubin (mg/dL)	0.18 ± 0.02	1.1 ± 0.1*	2.2 ± 0.13**	2.3 ± 0.15**	2.4 ± 0.18**

The values are expressed as mean ± SEM (*n* = 6)

*ALP* alkaline phosphatase, *ALT* alanine transaminase, *AST* aspartate transaminase

*p < 0.05; **p < 0.01; ***p < 0.001, vs. the sham group

### Expression of proinflammatory cytokines in the brain and blood of BDL rats

**Fig. 2:**
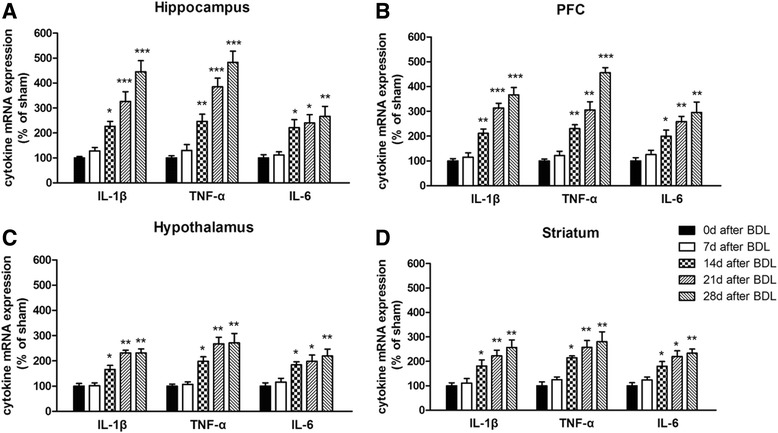
mRNA expression of TNF-α, IL-1β, and IL-6 in the hippocampus (**a**), cerebral cortex (**b**), hypothalamus (**c**), and striatum (**d**) of BDL animals in different days (0, 7, 14, 21, and 28 days) after surgery. Data are expressed as mean ± SEM (*n* = 6). *p < 0.05, **p < 0.01, and ***p < 0.001, when compared to the sham group

To investigate the time course and regional response of proinflammatory cytokine expression following BDL surgery, messenger RNA (mRNA) levels of IL-1β, IL-6, and TNF-α in the hippocampus, cerebral cortex, hypothalamus, and striatum of each rat were measured 0, 7, 14, 21, and 28 days after BDL surgery. mRNA levels of TNF-α, IL-1β, and IL-6 in the hippocampus of BDL rats were significantly elevated relative to the sham group 14 days after BDL surgery (*p* < 0.05, *p* < 0.01, Fig. 2a). Similar increases of TNF-α, IL-1β, and IL-6 mRNA expression levels were also found in the cerebral cortex, hypothalamus, and striatum (*p* < 0.05, *p* < 0.01, Fig. 2b–d).

**Fig. 3:**
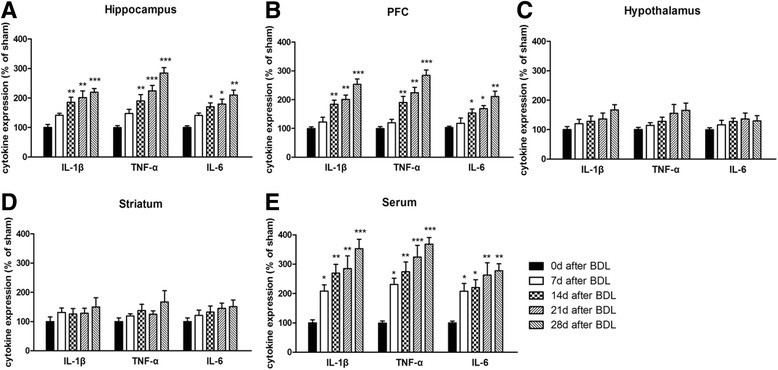
Expression of TNF-α, IL-1β, and IL-6 in the hippocampus (**a**), cerebral cortex (**b**), hypothalamus (**c**), striatum (**d**), and serum (**e**) of BDL animals in different days (0, 7, 14, 21, and 28 days) after surgery. Data are expressed as mean ± SEM (*n* = 6). *p < 0.05, **p < 0.01, and ***p < 0.001, when compared to the sham group

To confirm the regional response of proinflammatory cytokine expression after BDL, the protein levels of IL-1β, IL-6, and TNF-α of each rat were also measured by ELISA. As shown in Fig. 3a, in the hippocampus, IL-1β, IL-6, and TNF-α levels were significantly increased after BDL surgery [*F*(5,29) = 10.41, *p* < 0.001, for IL-1β; *F*(5,29) = 9.44, *p* < 0.001, for IL-6; *F*(5,29) = 17.93, *p* < 0.001, for TNF-α]. These increases were more obvious as time went on. Similarly, BDL surgery caused increases of IL-1β, IL-6, and TNF-α levels in the cerebral cortex [*F*(5,29) = 17.12, *p* < 0.01, for IL-1β; *F*(5,29) = 9.15, *p* < 0.001, for IL-6; *F*(5,29) = 21.3, *p* < 0.01, for TNF-α]. However, no significant differences of IL-1β, IL-6, and TNF-α expression were observed in the hypothalamus and striatum (Fig. 3c, d).

Likewise, we measured the protein level of proinflammatory cytokines in the blood of each rats by ELISA and found that the expression of IL-1β, IL-6, and TNF-α obviously increased 7 days after BDL [*F*(5,29) = 10.32, *p* < 0.001, for IL-1β; *F*(5,29) = 6.437, *p* < 0.01, for IL-6; *F*(5,29) = 14.31, *p* < 0.001, for TNF-α]. These increases were more obvious as time went on (Fig. 3e).

### IDO expression in the brain of BDL rats

**Fig. 4:**
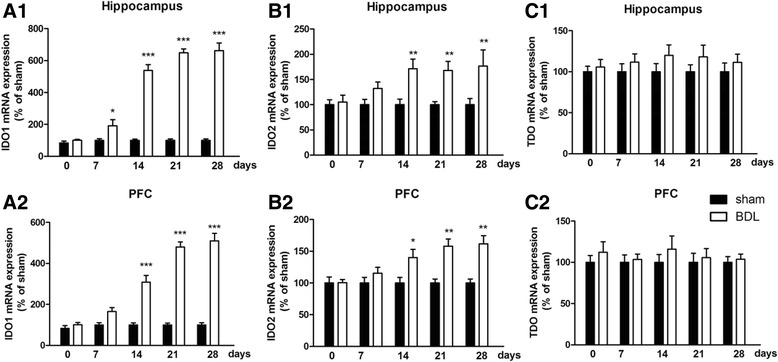
mRNA expression of IDO1 (**a**1), IDO2 (**b**1), and TDO (**c**1) in the hippocampus and mRNA levels of IDO1 (**a**2), IDO2 (**b**2), and TDO (**c**2) in the cerebral cortex of BDL animals in different days (0, 7, 14, 21, and 28 days) after surgery. Data are expressed as mean ± SEM (*n* = 6). *p < 0.05, **p < 0.01, and ***p < 0.001, when compared to the sham group

**Fig. 5:**
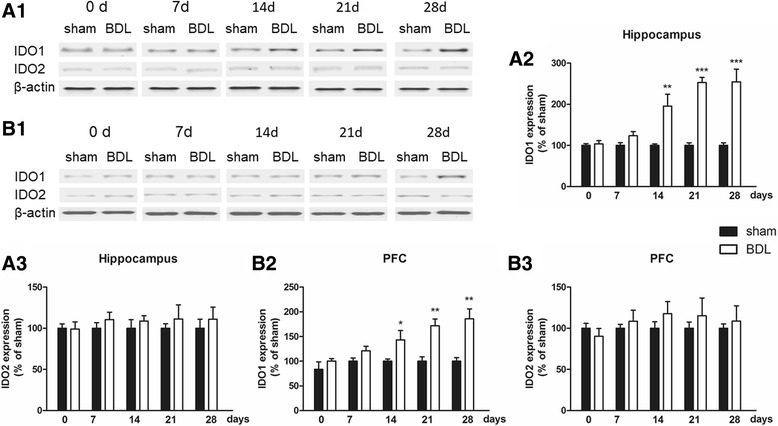
Expression of IDO1 and IDO2 in the hippocampus (**a**1) and cerebral cortex (**b**1) of BDL animals in different days (0, 7, 14, 21, and 28 days) after surgery. Analytical results of **a**1 are summarized in **a**2 and **a**3, and detailed results of **b**1 are summarized in **b**2 and **b**3. Data are expressed as mean ± SEM (*n* = 6). *p < 0.05, **p < 0.01, and ***p < 0.001, when compared to the sham group

To examine the role of IDO activity in the pathogenesis of HE, we measured both mRNA and protein levels of IDO1, IDO2, and TDO in the brain of each rat. For IDO is activated by proinflammatory cytokines, IDO activity was tested only in the hippocampus and cerebral cortex. As shown in Fig. 4, compared with the sham group, high IDO1 and IDO2 mRNA expression levels were observed in the hippocampus and cerebral cortex 14 days after BDL surgery (*p* < 0.001, *p* < 0.001, Fig. 4a–c), and these high expression levels last until the 28th day. Interestingly, for protein expression, only abnormal IDO1 level was found in these two brain regions from the 14th day to the 28th day (*p* < 0.001, Fig. 5).

### Effect of 1-MT on depressive-like behavior in BDL rats

**Fig. 6:**
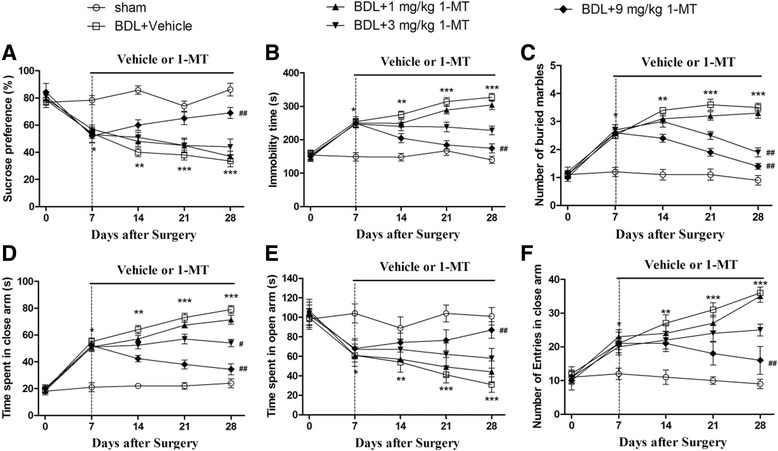
Effects of the IDO inhibitor 1-MT (1, 3, and 9 mg/kg) on the depressive-like behaviors (**a**, **b**) and anxiety behaviors (**c**–**f**) of BDL animals. Depressive-like behavior test: sucrose preference test (**a**) and forced swimming test (**b**); anxiety-like behavior test: marble-burying test (**c**) and elevated plus maze test (**d**–**f**). The time spent in closed arms (**d**), the time spent in open arms (**e**), and the number of entries in closed arms (**f**) assessed by elevated plus maze of BDL animals in different days (0, 7, 14, 21, and 28 days) after surgery. Data are expressed as mean ± SEM (*n* = 8). *p < 0.05, **p < 0.01, and ***p < 0.001, when compared to the sham group; ^#^*p* < 0.05 and ^##^*p* < 0.01, when compared to the BDL group

Effect of 1-MT on depressive-like behavior in BDL rats was assessed by the sucrose preference test and forced swimming test. BDL surgery induced a significant decrease in the preference of the sucrose solutions in the model group compared with the sham group (Fig. 6a). However, chronic treatment with 1-MT (1, 3, and 9 mg/kg) increased the sucrose preference, and the maximal effect was achieved when 9 mg/kg of 1-MT was treated for 21 days (*p* < 0.01).

As illustrated in Fig. 6, significant difference was found between groups in the FST (*p* < 0.001). Seven days after BDL surgery, the immobility time of these rats significantly increased when compared with the corresponding sham groups in the FST (*p* < 0.01, *p* < 0.001). However, treatment with 1-MT (dose of 9 mg/kg) abolished this adverse effect of BDL (*p* < 0.01, Fig. 6b).

### Effect of 1-MT on anxiety-like behavior in BDL rats

Effect of 1-MT on anxiety-like behavior in BDL rats was assessed by the marble-burying test and elevated plus maze test. BDL rats exhibited anxiety-like behavior compared with the sham rats (Fig. 6). In the marble-burying test, the increased number of buried marbles was observed in the BDL group (*p* < 0.001). Chronic administration with 1-MT prevented this increase, and this effect was found significant 21 days after 1-MT treatment (*p* < 0.01, Fig. 6c).

In the elevated plus maze test, BDL rats spent more time in the closed arms (*p* < 0.001, Fig. 6d) and less time in the open arms (*p* < 0.001, Fig. 6e). A significantly increased number of entries were observed in BDL group in the closed arms at the 7th day after surgery as compared with the sham group (*p* < 0.05, Fig. 6f). However, 1-MT prevented this adverse effect of BDL (*p* < 0.01).

### Effect of 1-MT on learning and memory function in BDL rats

**Fig. 7:**
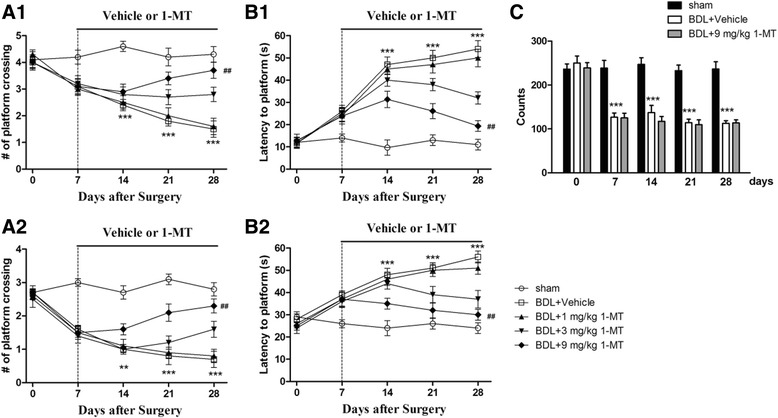
Effects of the IDO inhibitor 1-MT (1, 3, and 9 mg/kg) on memory deficits (**a**, **b**) and locomotor activity (**c**) induced by BDL in rats. Chronic treatment with 1-MT improves learning and memory behaviors in the Morris water maze task during the 1-h (**a**1, **a**2) or 24-h (**b**1, **b**2) test trials. Locomotor activity was assessed by the total number of counts recorded for 10 min in BDL animals in different days (0, 7, 14, 21, and 28 days) after surgery. Data are expressed as mean ± SEM (*n* = 8). **p < 0.01 and ***p < 0.001, when compared to the sham group; ^##^*p* < 0.01, when compared to the BDL group

Effect of 1-MT on learning and memory function in BDL rats was assessed by the Morris water maze test. As illustrated in Fig. 7, in the first probe trial (1 h after training), BDL rats needed longer latency to reach the platform position (*p* < 0.001 for the 14 days group) and a fewer number of crossings over the platform position compared with sham rats (*p* < 0.001 for 14 days after BDL). 1-MT-treated rats (9 mg/kg) took significantly less time to reach the platform position and took more crossings over the platform than model rats (*p* < 0.01, *p* < 0.01, Fig. 7a).

The memory retention for the platform location of rats was tested in the second probe trial (24 h after training). BDL rats showed significantly longer latency to reach the platform (*p* < 0.001 for the 14 days group) and fewer platform crossings compared with sham rats (*p* < 0.01 for the 14 days group). 1-MT treatment for 21 days reversed these phenomena (*p* < 0.01, Fig. 7b).

### Effect of 1-MT on spontaneous locomotor activity in BDL rats

Locomotor activity was assessed in terms of the number of photo beam counts recorded for 10 min. As shown in Fig. 7, BDL rats showed impairment in locomotor activity (48%) starting from day 7 till day 28 after BDL surgery when compared with sham rats. No significant difference was observed between BDL rats and BDL + 1-MT rats, when compared with sham rats [Fig. 7c, Additional file 1: Figure S1 for 1-MT (1 and 3 mg/kg) treatment].

### Effects of 1-MT on BDL surgery-induced expression changes in the neurotoxic kynurenine pathway of tryptophan metabolism

**Fig. 8:**
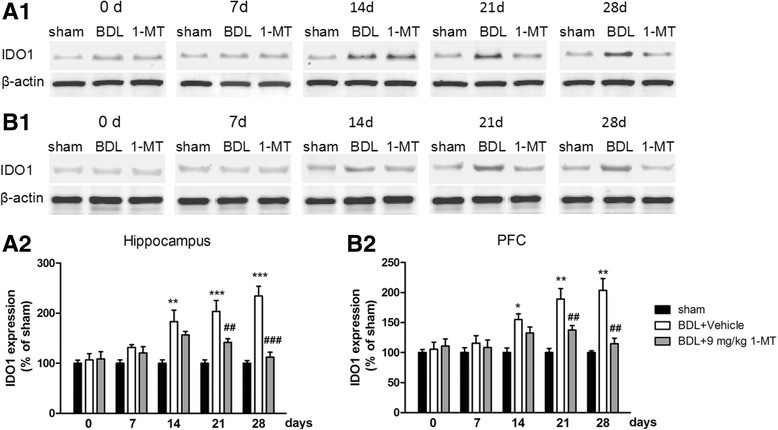
Effects of the IDO inhibitor 1-MT (9 mg/kg) on IDO1 expression in the hippocampus (**a**1) and cerebral cortex (**b**1) in different days (0, 7, 14, 21, and 28 days) after BDL surgery. Analytical results of **a**1 are summarized in **a**2, and detailed results of **b**1 are summarized in **b**2. Data are expressed as mean ± SEM (*n* = 6). *p < 0.05, **p < 0.01, and ***p < 0.001, when compared to the sham group; ^##^*p* < 0.01 and ^###^*p* < 0.001, when compared to the BDL group

**Fig. 9:**
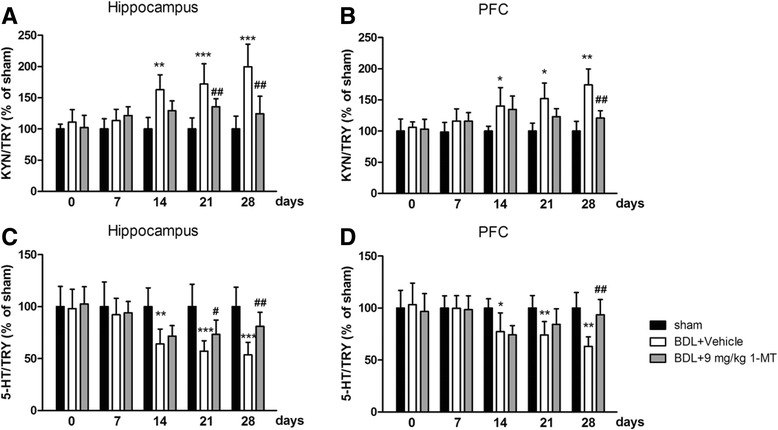
Effects of the IDO inhibitor 1-MT (9 mg/kg) on the kynurenine (KYN)/tryptophan (TRY) ratio in the hippocampus (**a**) and cerebral cortex (**b**) and on the serotonin (5-HT)/TRY ratio in the hippocampus (**c**) and cerebral cortex (**d**) of BDL rats. Data are expressed as mean ± SEM (*n* = 6). *p < 0.05, **p < 0.01, and ***p < 0.001, when compared to the sham group; ^#^*p* < 0.05 and ^##^*p* < 0.01, when compared to the BDL group

To examine the role of IDO1 activity in TRY metabolism in the pathogenesis of HE, we firstly measured the level of IDO1 and then tested expression levels of TRY, 5-HT, and KYN in the hippocampus and cerebral cortex using HPLC and subsequently determined the ratio of 5-HT or KYN to TRY. 1-MT (9 mg/kg) treatment reversed IDO1 increases in BDL rats [*p* < 0.001, *p* < 0.01, Fig. 8; Additional file 2: Figure S2 and Additional file 3: Figure S3 for 1-MT (1 and 3 mg/kg) treatment]. Furthermore, the ratios of KYN/TRY increased in the hippocampus and cerebral cortex of the BDL model group (*p* < 0.001, Fig. 9a for the hippocampus; *p* < 0.01, Fig. 9b for the cerebral cortex), and the 5-HT/TRY ratios decreased in these two brain regions (*p* < 0.001, Fig. 9a for the hippocampus; *p* < 0.01, Fig. 9b for the cerebral cortex). However, these changes were reversed by 9 mg/kg 1-MT treatment [Fig. 9, Additional file 4: Figure S4 for 1-MT (1 and 3 mg/kg) treatment].

**Fig. 10:**
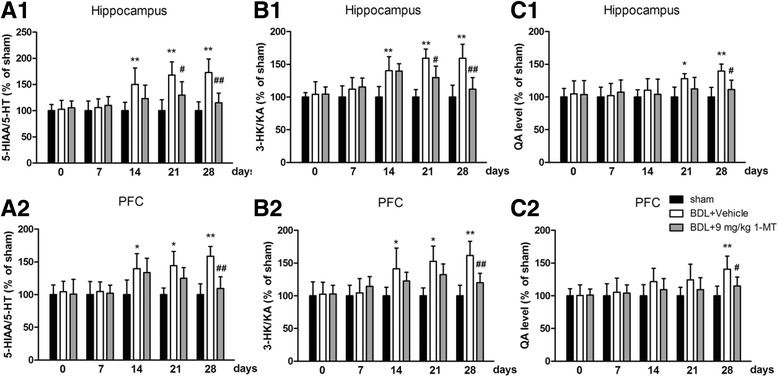
Effects of the IDO inhibitor 1-MT (9 mg/kg) on the 5-hydroxyindoleacetic acid (5-HIAA)/5-HT ratio, 3-hydroxykynurenine (3-HK)/kynurenic acid (KA) ratio, and quinolinic acid (QA) levels in the hippocampus (**a**1, **b**1, **c**1) and cerebral cortex (**a**2, **b**2, **c**2) of BDL rats. Data are expressed as mean ± SEM (*n* = 6). *p < 0.05 and **p < 0.01, when compared to the sham group; ^#^*p* < 0.05 and ^##^*p* < 0.01, when compared to the BDL group

In further study, we tested the levels of 5-HT metabolite (5-HIAA) and KYN metabolites (3-HK, KA, and QA) in the hippocampus and cerebral cortex by HPLC and found the 5-HIAA/5-HT ratio and 3-HK/KA ratio increased 28 days after BDL surgery (*p* < 0.01 for the hippocampus, *p* < 0.01 for the cerebral cortex, Fig. 10a, b). Moreover, high levels of QA were also observed 28 days after BDL surgery (*p* < 0.01 for the hippocampus, *p* < 0.05 for the cerebral cortex, Fig. 10c). However, all these increases induced by BDL were reversed by 9 mg/kg 1-MT treatment [Fig. 10, Additional file 5: Figure S5 and Additional file 6: Figure S6 for 1-MT (1 and 3 mg/kg) treatment].

## Discussion

The main findings of this study are as follows: BDL rats present the increases of anxiety-like and depressive-like behaviors, as well as memory deficits, and these behavioral changes are associated with the increased proinflammatory cytokine expression levels in the hippocampus and cerebral cortex, not in the hypothalamus and striatum; the KYN pathway of TRY metabolism may play an important role in inflammation-induced behavioral changes in HE; indoleamine-2,3-dioxygenase (IDO) inhibitor 1-methyl-l-tryptophan (1-MT) can reverse BDL-induced behavioral changes in rats, indicating that 1-MT may serve as a therapeutic agent for HE.

The BDL rat model is considered as a good experimental model for chronic HE [36, 37]. Seven days after biliary obstruction, the activity of liver marker enzymes (ALP, ALT, AST) and the levels of ammonia and bilirubin were significantly enhanced. We also observed significant increases of proinflammatory cytokines (TNF-α, IL-1β, and IL-6) in the hippocampus and cerebral cortex of BDL rats 3 weeks after biliary obstruction, which is similar to the results obtained by Hernandez-Rabaza et al. [9]. Curiously, increases of proinflammatory cytokines in the blood were observed 2 weeks after surgery, earlier than those in the brain. In agreement with the present data, Dadsetan’s research found that a high level of inflammation first appeared in the peripheral system in a HE animal model [38]. In fact, most of neuroinflammation was induced by peripheral inflammation [39, 40]. Initial neuroinflammatory response may be part of an adaptive beneficial process. However, chronic neuroinflammation results in adverse neurological consequences, such as disruption of neurotransmission and alteration of metabolic pathways [41–43], leading to many central nervous system diseases (depression, anxiety, and Alzheimer’s disease). Taken together, it seems plausible to hypothesize that these proinflammatory cytokines can be described as important features in HE development.

**Fig. 11:**
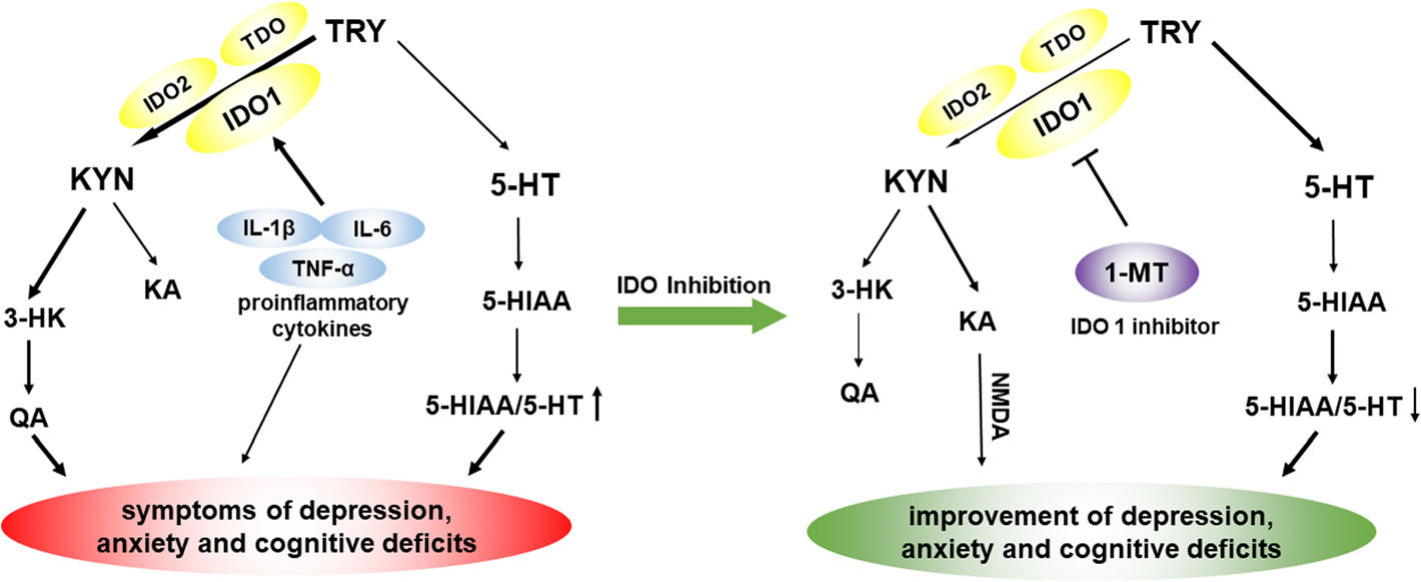
Pathways connecting tryptophan (TRY) metabolism to behavioral outcomes. IDO in the periphery and the central nervous system is activated by proinflammatory cytokines (e.g., TNF-α, IL-6, IL-1β). IDO activation results in decreased 5-HT/TRY, which induces the decrease in the ratio of 5-HIAA/5-HT, triggering individual symptoms of depression and anxiety. IDO activation is followed by the increased KYN/TRY ratio, which induces the imbalance of KYN metabolism. Normally, KYN is metabolized to kynurenic acid (KA), an NMDA antagonist. When IDO is activated, KYN is more likely metabolized to quinolinic acid (QA), a potent *N*-methyl-d-aspartate (NMDA) receptor agonist and may be a key contributor to increased neurotoxicity and cognitive deficits

The KYN pathway of TRY metabolism was hypothesized to be important in mediating the effects of proinflammatory cytokines on the brain [44]. Proinflammatory cytokines upregulate the expression and activity of IDO (Fig. 11), the rate-limiting enzyme of the KYN pathway [12–14]. KYN is synthesized from TRY by three enzymes, IDO1, IDO2, and TDO. In this study, mRNA and protein expression levels of IDO1 increased in the hippocampus and cerebral cortex after BDL, which is in conformity with the previous study [45]. However, as for IDO2, only mRNA level increased in the hippocampus and cerebral cortex. In fact, IDO2 is a more recently discovered homolog of IDO with much lower enzymatic activity compared with IDO1 [46], and previous studies demonstrated that proinflammatory stimuli only increase the mRNA level of IDO2 or have no impact on IDO2 expression [47, 48]. TDO expression did not increase in any brain region at any time point in response to BDL surgery. Overall, these data confirmed that IDO1 may be the only rate-limiting enzyme of KYN pathway upregulated in response to BDL.

In the present study, l type 1-MT, not 1-methyl-d-tryptophan (d type 1-MT), was employed to further explore the role of IDO1 activity in the change of behavior. In the present BDL model, depressive-like and anxiety-like behaviors were seen to accompany the increase of proinflammatory cytokines. Clinical depressive behavior results from a perceived absence of control over the outcome of a situation, which has been described as the learned helplessness theory [49]. Force swimming test, which was used to study animals with depression, was established based on this theory since helpless behaviors are frequently observed in depressed patients [49, 50]. Our data showed that the immobility times of BDL rats increased significantly when compared with the sham group. However, 1-MT treatment in the rats reversed these deficits. Another main symptom of depression is anhedonia, the inability to perform rewarded behaviors. The sucrose preference test is established based on this theory [51]. We observed that BDL surgery induced a significantly reduced sucrose preference in rats, while the IDO inhibitor 1-MT treatment reversed this decrease, indicating IDO may play an important role in depression accompanied by HE.

The elevated plus maze test and marble-burying test have been reported to be the most widely used tests for determining anxiety in animals. In the present study, BDL rats exhibited a decrease in the time spent and the number of open arm entries in the elevated plus maze test and also showed behavioral deficits in the marble-burying test. However, these symptoms were reversed by 1-MT treatment, indicating that anxiety behavior of BDL rats may be related to IDO upregulation. Some earlier researches confirmed our results: BDL rats spent less time in the open arms and more time in the closed arms of the maze as compared to the control animals [52, 53]. Furthermore, Souza et al.’s study had shown that 1-MT may process anti-anxiety-like action in neurodegenerative disorder [54].

Memory dysfunction is one of the main symptoms of HE patient. In the present study, this symptom was confirmed in BDL rats in the Morris water maze test. BDL rats needed longer latency to reach the platform and a decreased number of crossings over the previous platform from day 7 till the end of the experiment. Huang and his team had also shown that the latency to reach the platform which is used as a measure of spatial memory was longer in the case of BDL rats as compared to sham rats [55]. Furthermore, a recent study found that BDL rats took more time to locate the hidden platform than the control rats [56]. Our results also showed that 1-MT-treated BDL rats had restored memory performance and learned faster than non-treated rats, suggesting IDO regulation may be related to the memory deficits induced by BDL surgery. Corroborating our results, 1-MT treatment can ameliorate the memory deficit induced by amyloid-β1-42 peptide in mice [54].

Locomotor activity has been considered to be an index of wakefulness or alertness of mental activity, and the decrease of this activity may lead to sedation as a result of inhibition of the central nervous system [53]. Our data showed a significant decline in the locomotor activity from day 7 after rats received BDL surgery, and this effect continued till day 28. A similar result was also found in Leke et al.’s study [57]. In our data, no significant difference of locomotor activity was found between the BDL + 1-MT group and BDL group when compared with the sham group, suggesting 1-MT did not influence the accuracy of behavioral results.

To examine the role of IDO1 activity in TRY metabolism in the pathogenesis of HE, we measured the levels of TRY, 5-HT, and KYN in the hippocampus and cerebral cortex using HPLC. 5-HT and KYN are two major TRY metabolites produced through the regulation of metabolic enzymes, including IDO. 5-HT, one of the neurotransmitters, is thought to produce feelings of calmness, relaxation, and contentment. According to the monoamine hypothesis of depression and anxiety, if the amount of 5-HT is reduced, depleted, or dysfunctional for some reason, as in the case of decreased concentrations of TRY, these disorders will ensue. Recently, the low TRY level, as well as abnormal IDO expression, was observed in depression and anxiety animal models [13, 58]. Furthermore, IDO activation is associated with decreased 5-HT content and increased KYN content. Contemporary research also reveals that the relative balance of these opposing metabolic branches, rather than simply changes in the levels of individual metabolites, constitutes the pathogenic potential of kynurenine metabolism [59]. Curiously, the increased KYN/TRY ratio and decreased 5-HT/TRY ratio were observed in our study, indicating the balance of TRY metabolites was shifted from 5-HT synthesis to KYN formation in the brain of BDL rats. Moreover, 1-MT treatment reversed this imbalance of TRY metabolites and increased the 5-HT/5-HIAA ratio, which partly explained the antidepressant and anxiolytic-like effects of 1-MT in BDL rats.

The metabolism of KYN is physically compartmentalized within the brain [20, 51]. Normally, KYN is mainly metabolized to KA, not to 3-HK. During inflammation state, KYN is more likely to be metabolized to 3-HK, leading to the potential formation of several neurotoxic metabolites [60, 61]. Our data found that 3-HK increased, KA decreased, and the 3-HK/KA ratio increased in the brain of BDL rats, and these changes were normalized by 1-MT treatment. Furthermore, BDL treatment caused a significant change of QA, one of 3-HK’s neurotoxic metabolites and also an NMDA receptor agonist which is often used to induce memory impairment in animal models.

Despite the interesting findings, our study contains limitation. For these animals were weak after BDL surgery, additional intracerebral injection may cause high operative mortality. Thus, 1-MT was intraperitoneally injected in this study. However, it brings the issue that the neuroprotective effects observed after 1-MT injection may also be due to an indirect effect whereby the IDO1 inhibitor 1-MT alleviated the liver damage in addition to the direct action of IDO1 in the brain. We tested liver enzymes in serum after 1-MT treatment as a supplementary measurement. Data in Additional file 7: Table S1 indicated that 1-MT improved the liver function damaged by BDL surgery. Thus, in this study, 1-MT presented a hepatoprotective effect and this effect may influence the behavioral changes in HE rats. Nevertheless, it is reasonable to speculate that 1-MT exerted its protective effect mainly in the brain. Among the homologs of IDO, TDO is mainly expressed in the liver and IDO1 is mainly expressed in extrahepatic tissues, and 1-MT is specific for IDO1. Furthermore, previous studies reporting the potential therapeutic effect of 1-MT on diseases of the central nervous system administrated this inhibitor via i.p. injection or s.c. injection, because 1-MT can penetrate the blood-brain barrier [14, 62]. Detailed mechanism regarding how was the central nervous system affected by peripheral cytokines and to what extent it was affected remains to be explored in our future study.

## Conclusions

In summary, we found that behavioral impairment in BDL rats is associated with the increased proinflammatory cytokines in the brain. The current study firstly explored the role of the KYN pathway of TRY metabolism in the behavioral changes in BDL rats. We also demonstrated that the IDO1 inhibitor 1-MT can effectively alleviate BDL-induced behavioral changes in HE rats, suggesting that 1-MT was a possible candidate for the treatment of HE.

## Additional files

Additional file 1: Figure S1. Effects of IDO inhibitor 1-MT (1, 3 mg/kg) on locomotor activity induced by BDL in rats. Locomotor activity was assessed by total number of counts recorded for 10 min in BDL animals in different days (0d, 7d, 14d, 21d and 28d) after surgery. Data are expressed as mean ± SEM (*n* = 8). ***p < 0.001 when compared to the sham group. (TIFF 3600 kb)

Additional file 2: Figure S2. Effects of IDO inhibitor 1-MT (1, 3 mg/kg) on IDO1 expression in the hippocampus in different days (0d, 7d, 14d, 21d and 28d) after BDL surgery. Data are expressed as mean ± SEM (*n* = 6). ***p < 0.001 when compared to the sham group, #*p* < 0.05 when compared to the BDL group. (TIFF 3600 kb)

Additional file 3: Figure S3. Effects of IDO inhibitor 1-MT (1, 3 mg/kg) on IDO1 expression in the cerebral cortex in different days (0d, 7d, 14d, 21d and 28d) after BDL surgery. Data are expressed as mean ± SEM (*n* = 6). *p < 0.05 and **p < 0.01 when compared to the sham group, #*p* < 0.05 when compared to the BDL group. (TIFF 3600 kb)

Additional file 4: Figure S4. Effects of IDO inhibitor 1-MT (1, 3 mg/kg) on KYN/TRY ratio in the hippocampus (A) and cerebral cortex (B), and 5-HT/TRY ratio in the hippocampus (C) and cerebral cortex (D) of BDL rats. Data are expressed as mean ± SEM (*n* = 6). *p < 0.05, **p < 0.01 and ***p < 0.001 when compared to the sham group, #*p* < 0.05 when compared to the BDL group. (TIFF 3600 kb)

Additional file 5: Figure S5. Effects of IDO inhibitor 1-MT (1, 3 mg/kg) on 5-HIAA/5-HT ratio and 3-HK/KA ratio in the hippocampus (A, C) and cerebral cortex (B, D) of BDL rats. Data are expressed as mean ± SEM (*n* = 6). *p < 0.05, **p < 0.01 and ***p < 0.001 when compared to the sham group, #*p* < 0.05 when compared to the BDL group. (TIFF 3600 kb)

Additional file 6: Figure S6. Effects of IDO inhibitor 1-MT (1, 3 mg/kg) on QA levels in the hippocampus (A) and cerebral cortex (B) of BDL rats. Data are expressed as mean ± SEM (*n* = 6). *p < 0.05, **p < 0.01 and ***p < 0.001 when compared to the sham group. (TIFF 3600 kb)

Additional file 7: Table S1. Effect of 28 days 1-MT treatment on liver function tests in sham and BDL rats. (DOCX 17 kb)
