# Supplementary figures and images for: Role of the indoleamine-2,3-dioxygenase/kynurenine pathway of tryptophan metabolism in behavioral alterations in a hepatic encephalopathy rat model
Source: J Neuroinflammation. 2018 Jan 4;15:3. doi: 10.1186/s12974-017-1037-9 (PMC5753541; doi:10.1186/s12974-017-1037-9)

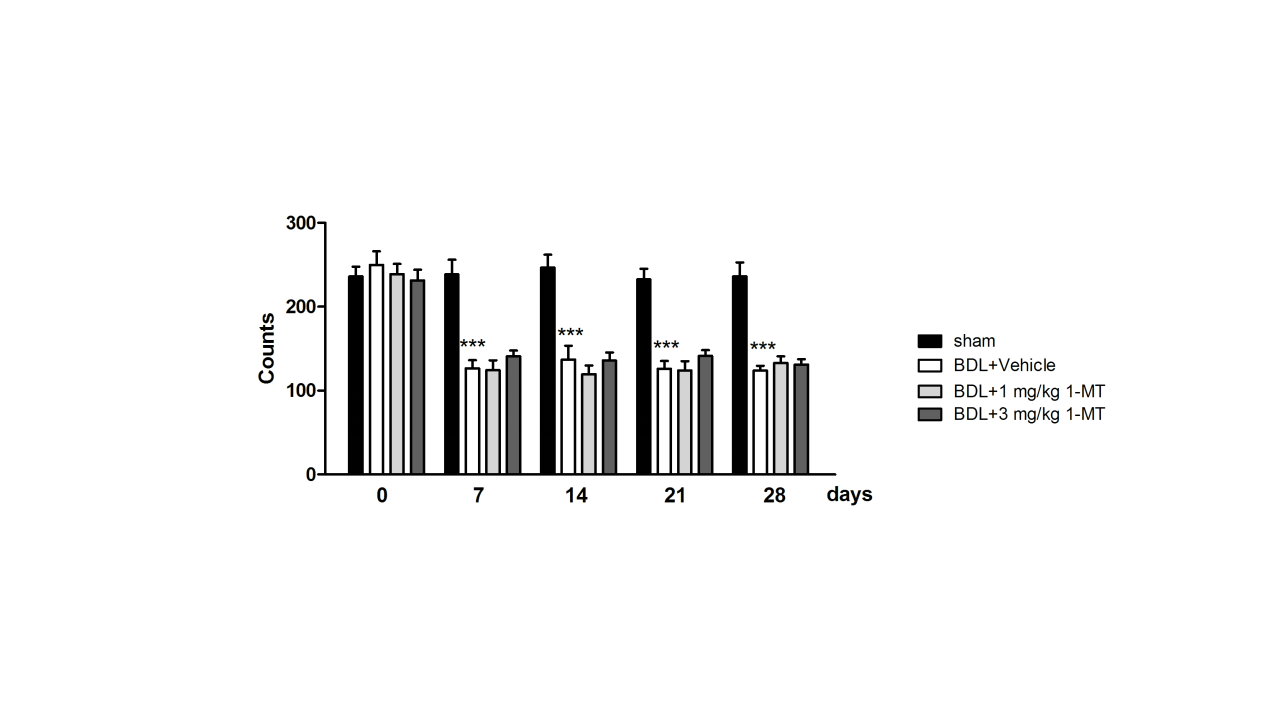

Supplement: Supplementary file 1 — Effects of IDO inhibitor 1-MT (1, 3 mg/kg) on locomotor activity induced by BDL in rats. Locomotor activity was assessed by total number of counts recorded for 10 min in BDL animals in different days (0d, 7d, 14d, 21d and 28d) after surgery. Data are expressed as mean ± SEM (n = 8). ***p < 0.001 when compared to the sham group. (TIFF 3600 kb) [file 12974_2017_1037_MOESM1_ESM.tif]

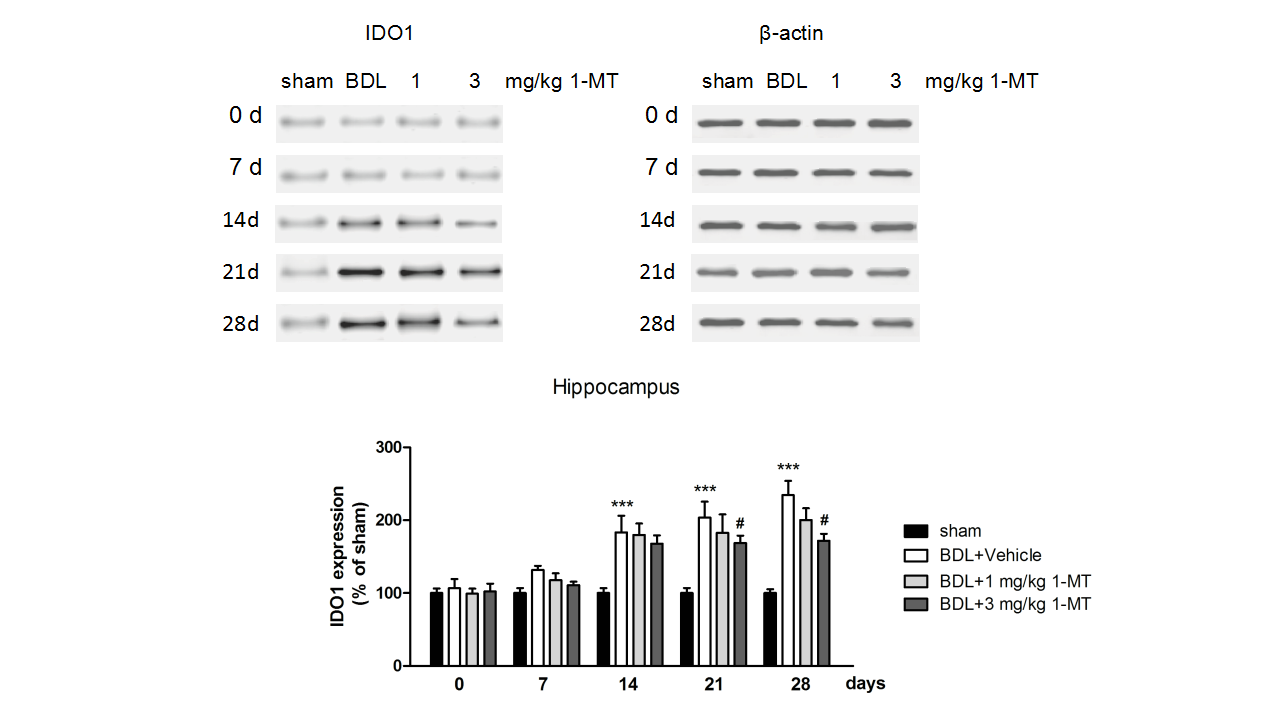

Supplement: Supplementary file 2 — Effects of IDO inhibitor 1-MT (1, 3 mg/kg) on IDO1 expression in the hippocampus in different days (0d, 7d, 14d, 21d and 28d) after BDL surgery. Data are expressed as mean ± SEM (n = 6). ***p < 0.001 when compared to the sham group, #p < 0.05 when compared to the BDL group. (TIFF 3600 kb) [file 12974_2017_1037_MOESM2_ESM.tif]

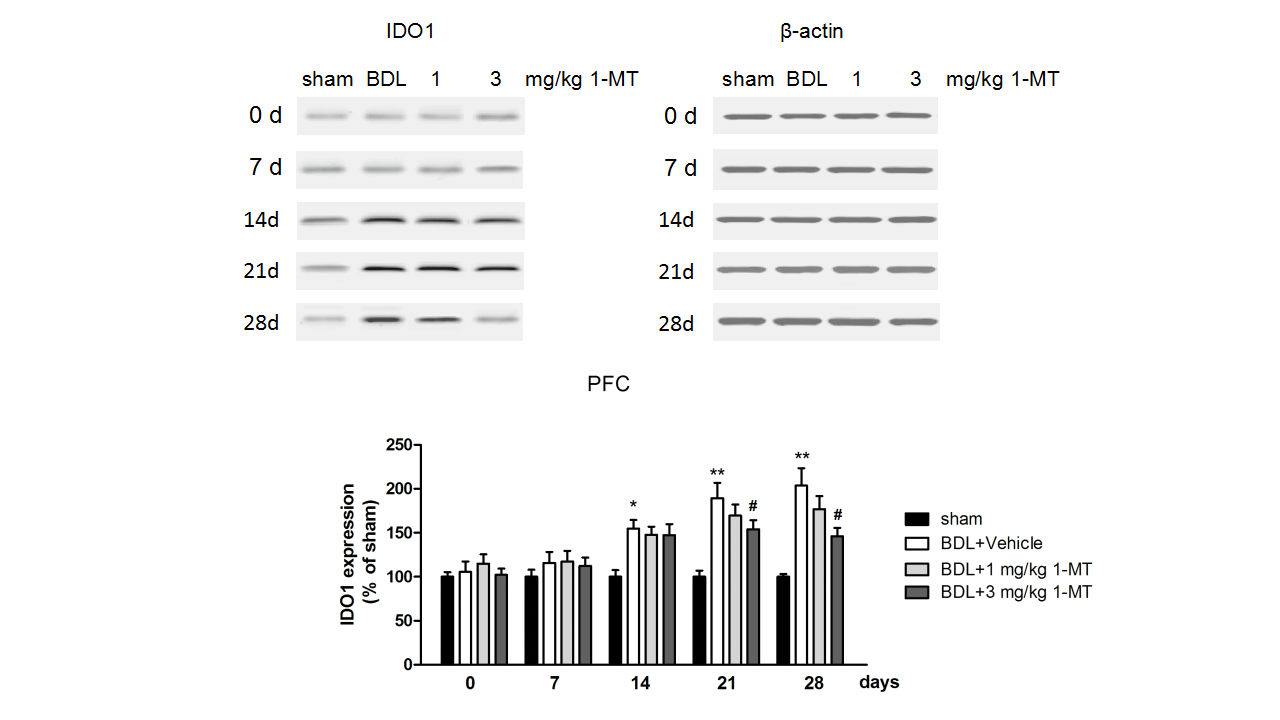

Supplement: Supplementary file 3 — Effects of IDO inhibitor 1-MT (1, 3 mg/kg) on IDO1 expression in the cerebral cortex in different days (0d, 7d, 14d, 21d and 28d) after BDL surgery. Data are expressed as mean ± SEM (n = 6). *p < 0.05 and **p < 0.01 when compared to the sham group, #p < 0.05 when compared to the BDL group. (TIFF 3600 kb) [file 12974_2017_1037_MOESM3_ESM.tif]

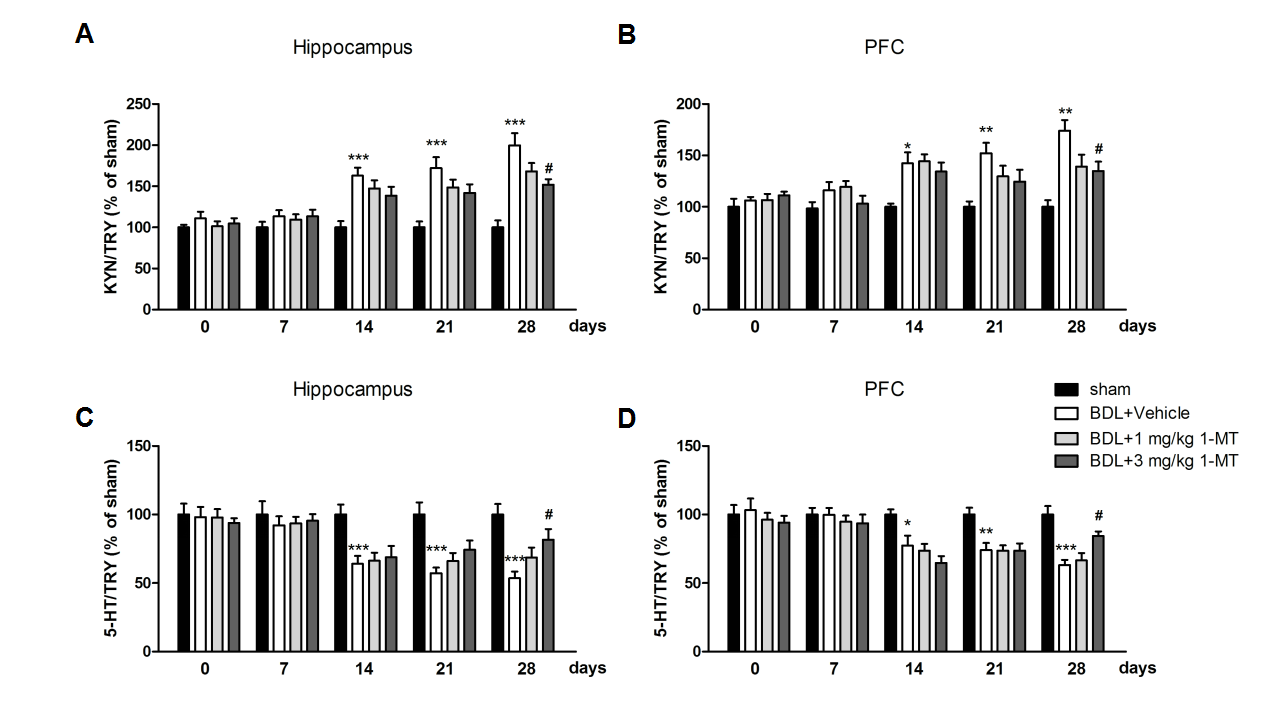

Supplement: Supplementary file 4 — Effects of IDO inhibitor 1-MT (1, 3 mg/kg) on KYN/TRY ratio in the hippocampus (A) and cerebral cortex (B), and 5-HT/TRY ratio in the hippocampus (C) and cerebral cortex (D) of BDL rats. Data are expressed as mean ± SEM (n = 6). *p < 0.05, **p < 0.01 and ***p < 0.001 when compared to the sham group, #p < 0.05 when compared to the BDL group. (TIFF 3600 kb) [file 12974_2017_1037_MOESM4_ESM.tif]

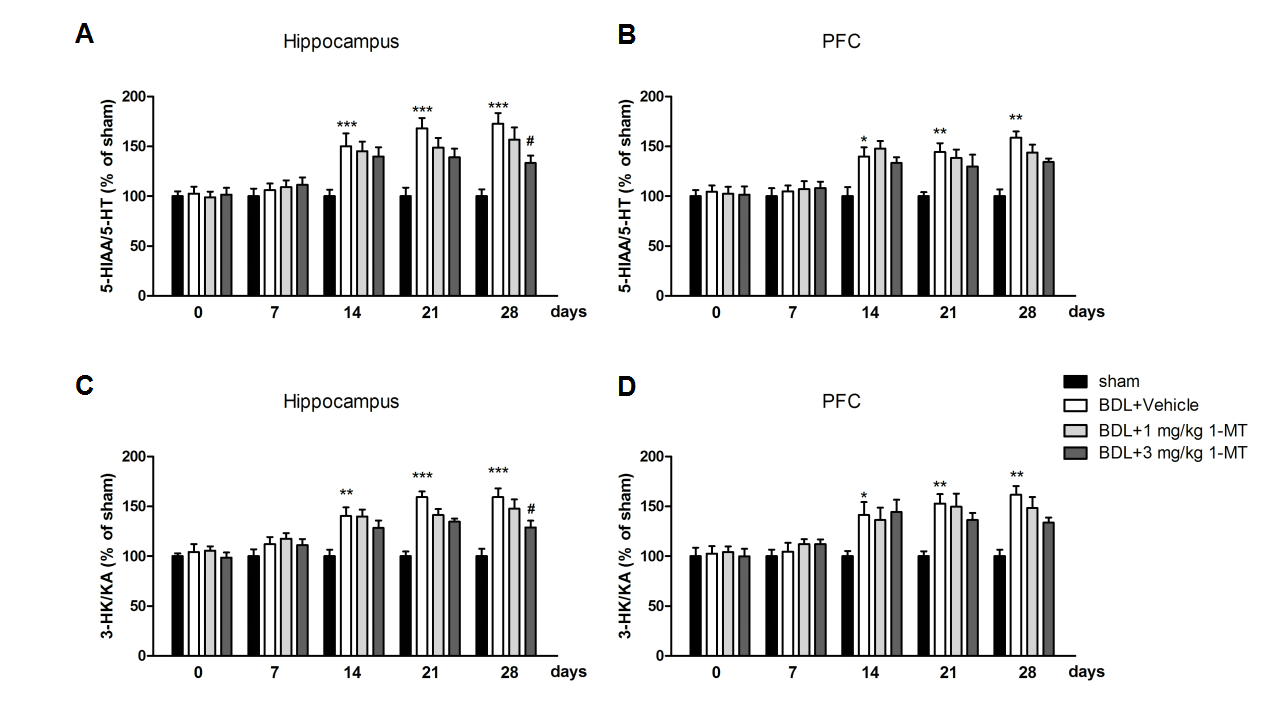

Supplement: Supplementary file 5 — Effects of IDO inhibitor 1-MT (1, 3 mg/kg) on 5-HIAA/5-HT ratio and 3-HK/KA ratio in the hippocampus (A, C) and cerebral cortex (B, D) of BDL rats. Data are expressed as mean ± SEM (n = 6). *p < 0.05, **p < 0.01 and ***p < 0.001 when compared to the sham group, #p < 0.05 when compared to the BDL group. (TIFF 3600 kb) [file 12974_2017_1037_MOESM5_ESM.tif]

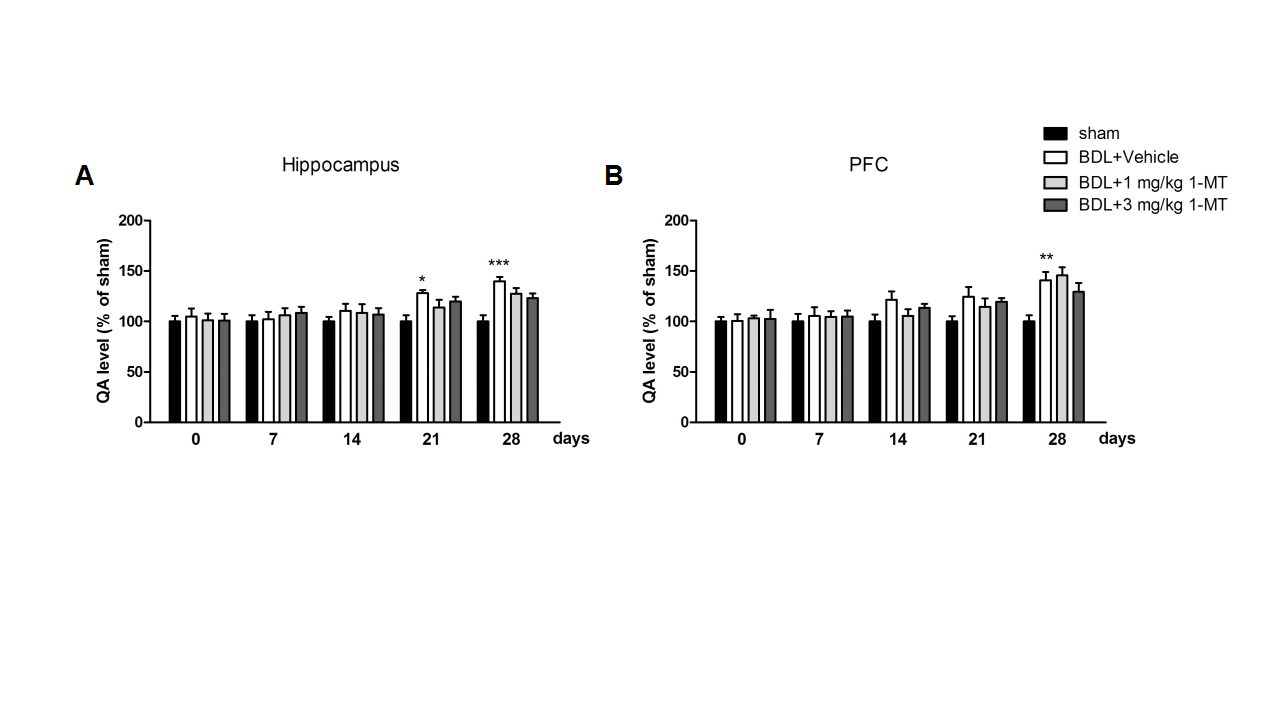

Supplement: Supplementary file 6 — Effects of IDO inhibitor 1-MT (1, 3 mg/kg) on QA levels in the hippocampus (A) and cerebral cortex (B) of BDL rats. Data are expressed as mean ± SEM (n = 6). *p < 0.05, **p < 0.01 and ***p < 0.001 when compared to the sham group. (TIFF 3600 kb) [file 12974_2017_1037_MOESM6_ESM.tif]
