# Supplementary material for: Role of the indoleamine-2,3-dioxygenase/kynurenine pathway of tryptophan metabolism in behavioral alterations in a hepatic encephalopathy rat model
Source: J Neuroinflammation. 2018 Jan 4;15:3. doi: 10.1186/s12974-017-1037-9 (PMC5753541; doi:10.1186/s12974-017-1037-9)
Supplement: Supplementary file 7 — Effect of 28 days 1-MT treatment on liver function tests in sham and BDL rats. (DOCX 17 kb) [file 12974_2017_1037_MOESM7_ESM.docx]

**Table S1 Effect of 28 days 1-MT treatment on liver function tests in sham and BDL rats.**

|  | Sham | BDL | BDL+1-MT  (1 mg/kg) | BDL+1-MT  (3 mg/kg) | BDL+1-MT  (9 mg/kg) |
| --- | --- | --- | --- | --- | --- |
| ALP (IU/L) | 110.4±6.9 | 312.1±11.5** | 301.2±11.3** | 264.2±14.3* | 195.3±15.7^#^ |
| ALT (IU/L) | 41.2±4.1 | 114.2±7.5** | 107.2±8.4** | 95.2±8.9** | 75.3±10.7^#^ |
| AST (IU/L) | 118.2±9.4 | 304.2±16.1** | 279.5±10.2** | 248.2±13.5** | 192.6±14.3* |
| Ammonia (μmol/L) | 19.2±4.1 | 191.2±11.3*** | 181.2±9.3*** | 131.2±10.5** | 103.4±9.2**^#^ |
| Total Bilirubin (mg/dL) | 0.44±0.02 | 6.1±0.33*** | 5.7±0.2*** | 3.1±0.3**^#^ | 2.1±0.5*^#^ |
| Direct Bilirubin (mg/dL) | 0.19±0.01 | 2.5±0.24*** | 2.5±0.3*** | 1.9±0.6** | 1.7±0.3** |
| Indirect Bilirubin (mg/dL) | 0.17±0.03 | 2.6±0.21** | 2.5±0.3** | 1.4±0.65* | 0.5±0.3*^#^ |

The values are expressed as mean ± S.E.M. n=6. * p < 0.05, ** p < 0.01 and ***p < 0.001 vs. sham group. ^#^ p < 0.05 vs. BDL group. ALP: Alkaline Phosphatase, ALT: Alanine Transaminase, AST: Aspartate Transaminase.
